# Assessing Diagnostic Precision: Adaptations of the Hopkins Symptom Checklist (HSCL-5/10/25) Among Tertiary-Level Students in Norway

**DOI:** 10.32872/cpe.13275

**Published:** 2024-12-20

**Authors:** Børge Sivertsen, Jens C. Skogen, Anne Reneflot, Marit Knapstad, Otto Robert Frans Smith, Leif Edvard Aarø, Benedicte Kirkøen, Bengt Oscar Lagerstrøm, Ann Kristin Skrindo Knudsen

**Affiliations:** 1 Department of Health Promotion, Norwegian Institute of Public Health, Bergen, Norway; 2 Department of Research & Innovation, Helse-Fonna HF, Haugesund, Norway; 3 Center for Alcohol & Drug Research, Stavanger University Hospital, Stavanger, Norway; 4 Centre for Evaluation of Public Health Measures, Norwegian Institute of Public Health, Oslo, Norway; 5 Department of Mental Health and Suicide, Norwegian Institute of Public Health, Oslo, Norway; 6 Department of Teacher Education, NLA University College, Bergen, Norway; 7 Department for Methodology and Data Collection, Statistics Norway, Oslo, Norway; 8 Department of Disease Burden, Norwegian Institute of Public Health, Bergen, Norway; Philipps-University of Marburg, Marburg, Germany

**Keywords:** depression, anxiety, students, young adults, questionnaires, psychometrics

## Abstract

**Background:**

Universities worldwide are witnessing a surge in mental health problems among students, particularly in anxiety and depression. The Hopkins Symptom Checklist (HSCL) is a popular screening tool, but its reliability in identifying mental disorders remains debated. The aim of this study was to evaluate the criterion validity of the HSCL-25, HSCL-10, and HSCL-5 using 30-day prevalence of major depressive episode (MDE) and generalized anxiety disorder (GAD) from a self-administered electronic version of the Composite International Diagnostic Interview, fifth version (CIDI 5.0), as the benchmark.

**Method:**

Data stem from a national survey targeting students in higher education in Norway. In a 2023 follow-up study on mental disorders, 5,568 participants completed both the HSCL-25 and the CIDI. Sex-specific optimal thresholds for all HSCL versions in relation to MDE and GAD (from CIDI) were determined using the Youden Index maximization.

**Results:**

The optimal cut-off values for detecting MDE or GAD with the HSCL-25 were 1.96 for males and 2.20 for females, displaying a good balance between sensitivity and specificity. Similar high and balanced sensitivity and specificity patterns were found for both the HSCL-10 and HSCL-5. However, all HSCL versions overestimated prevalence rates compared to the self-administered CIDI.

**Conclusions:**

All three HSCL versions showed high criterion validity. The data indicate that HSCL may be better as a screening tool than for precise estimation of MDE and GAD prevalence. For improved diagnostic accuracy, future HSCL versions should incorporate functional impairment assessment. This update would bring the HSCL into closer alignment with clinical diagnostic standards.

## Highlights

- This study assessed HSCL's ability to detect anxiety and depression in Norwegian college students.
- A self-administered version of CIDI was used as the gold standard for diagnostic accuracy.
- All three HSCL versions showed high criterion validity and good diagnostic precision.
- HSCL may be better as a screening tool than for precise estimation of mental disorders.

Universities and colleges worldwide are confronting a concerning rise in the incidence of mental health issues among their students, with projections significantly surpassing those observed in the general population (Ibrahim et al., 2013; McCloud et al., 2023). Recent systematic reviews on anxiety and depression have yielded a cumulative annual prevalence range of 25-30% among tertiary education students (Chi et al., 2023; Ibrahim et al., 2013; Kou et al., 2012; Sheldon et al., 2021).

Although diagnostic interviews maintain their status as the benchmark for mental disorder diagnosis (Nordgaard et al., 2013; Rettew et al., 2009), their demanding time and resource requirements hinder most researchers from incorporating comprehensive psychiatric interviews into their assessment battery. Consequently, a substantial portion of investigations are constrained to incorporating brief survey questionnaires when evaluating mental health issues (Auerbach et al., 2016). However, in order to obtain prevalence estimates that are as accurate as possible, it is important that the case-detection capabilities of these briefer questionnaires are thoroughly evaluated. Also, considering the changing trends and disparities observed across age cohorts and research populations, it is imperative to re-examine previously established cut-off values to ensure that the case-detection capabilities are effectively validated for each distinct study population.

In this context, a frequently employed survey instrument is the Hopkins Symptom Checklist (HSCL), initially devised during the 1950s as a clinical tool for assessing symptoms of several mental disorders. Although the original iteration encompassed an extensive array of mental disorders, one of the most commonly used versions today is the HSCL-25. This abbreviated version focuses on two symptom dimensions, anxiety and depression. However, the capacity of the HSCL-25 to differentiate between these two conditions across different sexes is not fully established (Sandanger et al., 1998; Skogen et al., 2017). Since the 1990s, the efficacy of HSCL-25 in identifying cases has been examined a few times through comparisons with structured diagnostic interviews, suggesting that while HSCL-25 performs adequately in detecting depression, the results for anxiety are more variable (Sandanger et al., 1998; Veijola et al., 2003).

The HSCL-25 uses a scoring range of 1 to 4, and to determine the commonly used mean HSCL-25 score, the total score is divided by the item count. A traditional cut-off value of 1.75 is commonly employed to indicate major depressive disorder (Glaesmer et al., 2014; Sandanger et al., 1998; Veijola et al., 2003). However, a recent Spanish study has challenged this one-size-fits-all approach, proposing distinct optimal cut-off values for women (1.76) and men (1.84) (Rodríguez-Barragán et al., 2021). This suggests that sex-specific thresholds might be necessary for more accurate diagnosis, considering the different ways in which men and women experience and report mental health symptoms.

Further abbreviated iterations of the HSCL have been developed, with both HSCL-5 and HSCL-10 now in widespread use. However, few studies have investigated the case-detection capability of these versions, and none have done so for males and females separately. To the best of our knowledge, just one study has directly contrasted HSCL-5 and HSCL-10 against a structured diagnostic interview. A recent Spanish study demonstrated good reliability and validity of both tools for detecting depression (Rodríguez-Barragán et al., 2023). Additionally, a recent Norwegian study assessed HSCL-5's capacity for identifying cases in the general population, and its findings indicate that HSCL-5 effectively identifies people with generalized anxiety disorder or major depressive disorder within this context (Kirkøen et al., Manuscript in preparation).

Based on these considerations, the objective of this current study is to assess the proficiency of HSCL-25, HSCL-10, and HSCL-5 in detecting cases of major depressive episode (MDE) and generalized anxiety disorder (GAD) in a national sample of college and university students. Our focus is on potential sex-specific cut-off values and differences, using a recently developed self-administered electronic version of the Composite International Diagnostic Interview (CIDI) 5.0. This approach aims to enhance the accuracy of case-detection and ensure that the nuanced mental health experiences of both sexes are adequately captured.

## Method

### Setting and Participants

The primary population for this study is derived from the Norwegian SHOT study (Students' Health and Wellbeing Study), a nationwide survey that centres on students pursuing higher education. Since 2010, four major surveys have been conducted, with the latest wave conducted in 2022. The SHOT2022 survey comprehensively explored a multitude of dimensions encompassing health and lifestyle. These dimensions included psychological distress, suicidality, life satisfaction, loneliness, sleep problems, sexual harassment, pain, physical exercise, alcohol and drug use, as well as demographic and educational parameters. Comprehensive information concerning the SHOT study has been previously documented (Sivertsen et al., 2019).

During the survey period, SHOT2022 was distributed electronically via a web-based platform and was open for submissions from February 8 to April 19, 2022. Invitations for participation were sent to all full-time Norwegian students engaged in higher education, both within the country and abroad. Extensive efforts were undertaken to increase awareness about the study through channels such as email, SMS, and informational campaigns conducted by welfare organizations and educational institutions. A total of 169,572 students met the study's inclusion criteria, which required them to be full-time college or university students and hold Norwegian citizenship. Of these, 59,544 students completed the online questionnaire after receiving two reminders. This resulted in a response rate of 35.1% (which did not differ between geographical regions). For the present study, the inclusion criteria specified that participants be between the ages of 18 and 35 years. Consequently, a subset of 53,362 students within this age range was selected for analysis. When consenting to participate in the SHOT2022, students were given the option to express their interest in participating in a follow-up study on mental disorders. Out of the total participants, 26,311 students consented to be a part of this follow-up study. To better reflect the sex distribution of the base study population, more male students were invited to participate in the CIDI study. Consequently, 16,418 students, officially registered as of January 2023, were invited. However, fewer male students consented to follow-up contact compared to females. This led to females comprising a higher proportion (70.4%) of the invitations for the CIDI study.

**Figure 1:**
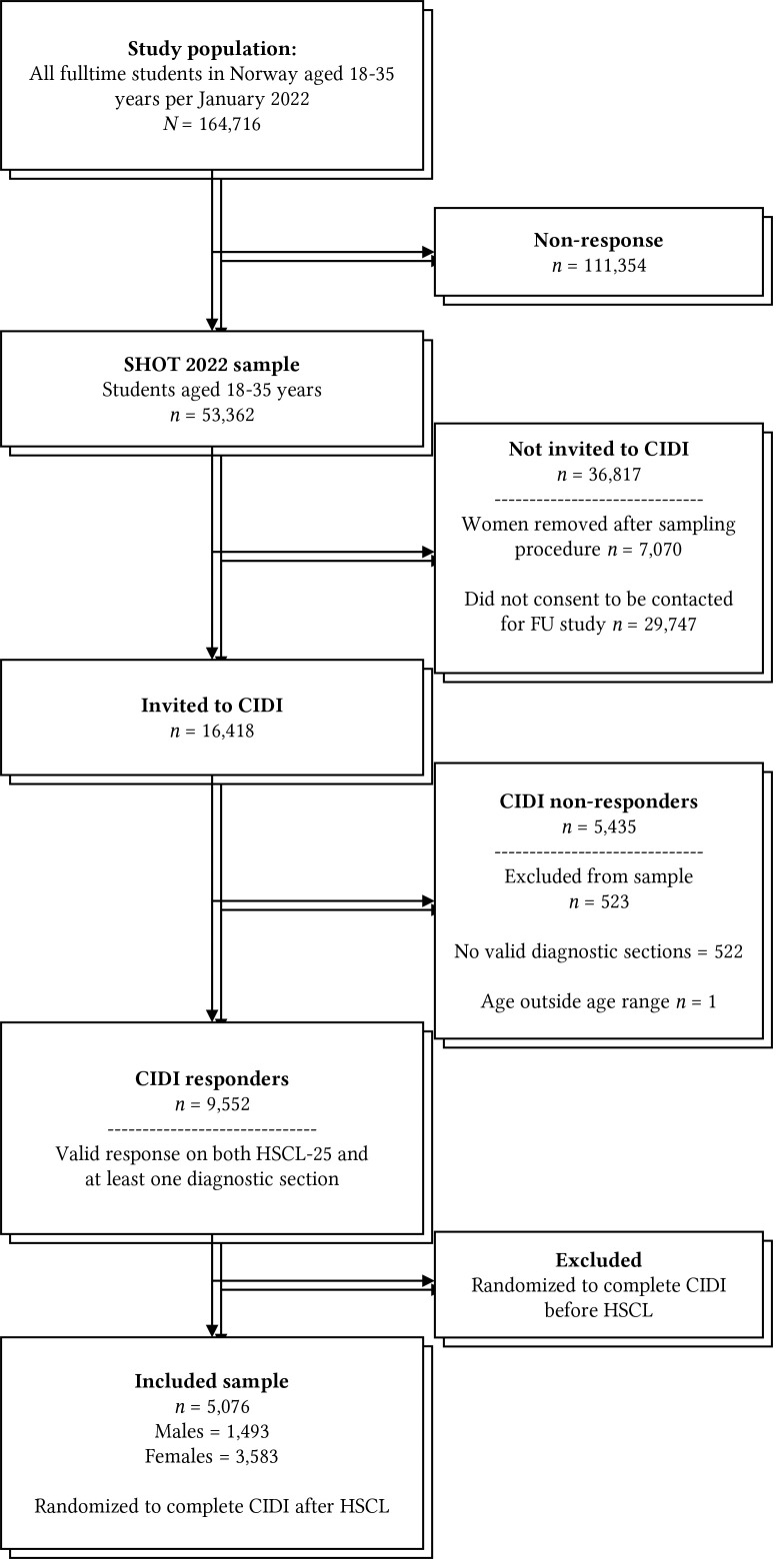
Participant Flow in the Study

Figure 1 illustrates the participation process for the current study. Out of 9,552 students who provided valid responses on both the HSCL-25 and at least one of the CIDI diagnostic sections, half were randomly selected to complete the HSCL-25 before the CIDI. This resulted in a subset of 5,076 participants who provided valid scores on the HSCL-25 and then completed the CIDI. The remaining half completed the assessments in the reverse order. This deliberate sequencing was chosen to facilitate future investigations into the potential influences of the order of questionnaire administration on the reported results from the HSCL-25.

For the purposes of the present study, we only used observations where the HSCL-25 was administered before the CIDI, as screening instruments are normally not preceded by full diagnostic assessments in population-based surveys. The CIDI study took place between January 24 and February 6, 2023, approximately 12 months after the SHOT2022 survey was conducted. More detailed information on the participation process has been published elsewhere (Sivertsen et al., 2023).

### Instruments

#### Sociodemographic Information

Participants' age and sex information were derived from their 11-digit Norwegian national identity numbers.

#### Depression and Anxiety Disorders: The CIDI

The data collection utilized a recently developed electronic, self-administered version of the Composite International Diagnostic Interview, fifth version (CIDI 5.0), developed for the World Health Organization (WHO) World Mental Health (WMH) Surveys (Kessler & Üstün, 2004). Both the original CIDI and this self-administered version maintained the same sequence of CIDI modules, and the wording of the questions remained nearly identical, though instructions were slightly adapted for self-administration. To reduce participant burden and enhance response rates, the current version excluded certain diagnostic categories, namely bipolar, obsessive-compulsive, PTSD, ADHD, and personality disorders. In short, CIDI 5.0 is a standardized interview that assesses 30-days, 12 months and lifetime prevalence for several mental and substance use disorders according to diagnostic criteria in the Diagnostic and Statistical Manual of Mental Disorders 5th edition (DSM-5) (American Psychiatric Association, 2013). The interview version of CIDI 3.0 has shown good concordance with diagnostic instruments such as the Structured Clinical Interview for DSM-IV (SCID) (Haro et al., 2006) and Schedules for Clinical Assessment in Neuropsychiatry (SCAN) (Jordanova et al., 2004).

Current mental disorder was defined as the presence of MDE or GAD during the 30 days before study participation. We also estimated prevalence rates for mental disorders spanning a 12-month period and over the lifetime, but these estimations were not incorporated into the present study, aligning with its specific emphasis on assessing current mental disorders in comparison with the HSCL. The operationalization of these diagnoses was based on algorithms developed for CIDI 5.0 in the WMH Surveys Initiative.

#### The Hopkins Symptom Checklist (HSCL)

Mental health problems in the 14 days before the survey were assessed by the widely used *Hopkins Symptom Checklist* (HSCL-25) (Derogatis et al., 1974), derived from the 90-item Symptom Checklist (SCL-90). The score for the HSCL is determined by taking the sum of the item scores and dividing it by the total number of items responded to, yielding a potential range of 1 to 4. An investigation of the factor structure based on the SHOT2014 dataset showed that a unidimensional model had the best psychometric properties in the student population and not the original subscales of anxiety and depression (Skogen et al., 2017). Details on the development of mental health problems assessed with the HSCL-25 in the SHOT waves were recently published by Knapstad and colleagues (Knapstad et al., 2021). The items included in the three different versions of the HSCL are listed in Table 1.

**Table 1:** Items Included in the Different HSCL Iterations^a^

Item content	HSCL-25	HSCL-10	HSCL-5
1. Suddenly scared for no reason	◯	◯	
2. Feeling fearful	◯	◯	◯
3. Faintness, dizziness, or weakness	◯	◯	
4. Nervousness or shakiness inside	◯		◯
5. Heart pounding or racing	◯		
6. Trembling	◯		
7. Feeling tense or keyed up	◯	◯	
8. Headaches	◯		
9. Spells of terror or panic	◯		
10. Feeling restless, can't sit still	◯		
11. Feeling low in energy, slowed down	◯		
12. Blaming yourself for things	◯	◯	
13. Crying easily	◯		
14. Loss of sexual interest or pleasure	◯		
15. Poor appetite	◯		
16. Difficulty falling asleep, staying asleep	◯	◯	
17. Feeling hopeless about the future	◯	◯	◯
18. Feeling blue	◯	◯	◯
19. Feeling lonely	◯		
20. Feeling trapped or caught	◯		
21. Worrying too much about things	◯		◯
22. Feeling no interest in things	◯		
23. Thoughts of ending your life	◯		
24. Feeling everything is an effort	◯	◯	
25. Feelings of worthlessness	◯	◯	

^a^Each symptom is rated on a four-point frequency scale (1 =  not at all, 2 = a little, 3  =  quite a lot, 4  =  extremely).

### Statistical Analyses

In the present study, we first present summary statistics for HSCL scores and the prevalence of MDE and GAD, stratified by sex (Table 3 and Table 4). Subsequently, we proceeded to determine sex-specific optimal cut-off points for HSCL-25, HSCL-10, and HSCL-5 in relation to either MDE or GAD combined, and for MDE and GAD separately, as identified through CIDI. This estimation was grounded in the maximization of the Youden Index, which is a commonly used metric for binary classification in validation studies aimed at striking a balance between sensitivity and specificity. The formula for the Youden Index, denoted as ('sensitivity' + 'specificity') - 1, yields a scale ranging from 0 to 1. Higher values signify better discriminative capacity, where 0 denotes no discrimination, and 1 reflects perfect discrimination. Although rules of thumb always must be considered in conjunction with other aspects, a score below 0.5 on the Youden Index indicates that the test in question may not be useful as a classification tool, whereas a score surpassing 0.5 can be construed as indicating a valuable test. Alongside the Youden Index, we also present a comprehensive view of overall accuracy, sensitivity, specificity, positive and negative predictive values (PPV and NPV), and the area under the curve (AUC). All analyses were performed using R 4.2.2.

## Results

As detailed in Table 2, the sample for the CIDI study primarily consisted of female students of Norwegian ethnicity, with an average age of 24 years, and about half were single. Most participants had parents with high educational levels. Compared with the overall SHOT2022 study, the sociodemographic characteristics were similar, except for a slightly higher proportion of females in the CIDI study (70%) compared to the SHOT2022 study (66%), as shown in Table 2. Non-respondents to the CIDI study, who were invited but did not participate, differed mainly in having parents with lower education levels.

**Table 2:** Demographical Characteristics and Representativeness of the CIDI Responders, CIDI Non-Responders and the Overall Sample Based on Data From 2022

Characteristic	CIDI responders (*n* = 9,552)	CIDI non-responders (*n* = 6,993)	*p* ^a^	SHOT2022^b^ (*n* = 53,362)	*p* ^a^
**Age, mean (*SD*)**	24.03 (3.28)	23.97 (3.24)	.24	23.98 (1.85)	.14
Sex, % (*n*)			.35		< .001
Women	70.0 (6,686)	71.0 (4,968)		66.4 (35,423)	
Men	30.0 (2,866)	29.0 (2,025)		33.6 (17,939)	
Marital status, % (*n*)			.20		.81
Single	51.3 (4,904)	50.4 (3,526)		51.0 (27,197)	
Boy-/girlfriend	22.5 (2,152)	23.7 (1,659)		22.8 (12,152)	
Cohabitant	22.6 (2,156)	22.4 (1,563)		22.6 (12,058)	
Married/registered partner	3.2 (308)	3.0 (207)		3.1 (1,667)	
*Missing*	*0.3 (32)*	*0.5 (38)*		*0.5 (288)*	
Maternal education, % (*n*)			.01		.27
Primary	4.3 (407)	5.3 (369)		4.5 (2,407)	
Secondary	27.2 (2,601)	27.6 (1,931)		27.6 (14,707)	
College/university	65.9 (6,290)	64.2 (4,488)		64.3 (34,326)	
*Missing*	*2.7 (254)*	*2.9 (205)*		*3.6 (1,992)*	
Paternal education, % (*n*)			.02		.39
Primary	5.7 (544)	6.8 (473)		6.0 (3,182)	
Secondary	34.9 (3,335)	35.0 (2,449)		35.1 (18,735)	
College/university	54.6 (5,211)	52.7 (3,687)		53.3 (28,446)	
*Missing*	*4.8 (462)*	*5.5 (384)*		*5.6 (2,999)*	
**HSCL-25, Mean (*SD*)**	1.88 (0.61)	1.90 (0.61)	.03	1.86 (0.59)	< .001
*Missing, % (n)*	*0.2 (17)*	*0.3 (24)*		*0.4 (214)*	

*Note.* SHOT2022 = Students’ Health and Wellbeing Study 2022; CIDI = Composite International Diagnostic Interview; HSCL-25 = Hopkins Symptoms Checklist – 25 items version.

^a^compared with the CIDI responders group (*p*-values based on Chi-squared test [categorical variables] or *t*-test [continuous variables]). ^b^grand mean for the SHOT2022 sample aged 18-35.

The level of mental health problems, measured by the HSCL-25 in the SHOT2022 study, was marginally lower in CIDI respondents (*M* = 1.88, *SD* = 0.61) compared to non-respondents (*M* = 1.90, *SD* = 0.61, Cohen’s *d* = 0.03). However, CIDI respondents had a slightly higher HSCL-25 score than the overall SHOT2022 sample (*M* = 1.86, *SD* = 0.57, Cohen’s *d* = 0.03).

Figure 1 details the participation process of the current study. A total of 5,076 participants completed both the CIDI and HSCL-25 in advance. The mental health characteristics of the sample are detailed in Table 3. Females reported considerably higher average scores on all HSCL iterations, and the prevalences of 30-day MDE and GAD were much higher among females as well, as detailed in Table 3.

**Table 3:** Mental Health Characteristics of the Study Sample

Characteristic	Males, *N* = 1,493	Females, *N* = 3,583	*p*
HSCL-25, *M* (*SD*)	1.67 (0.53)	1.96 (0.60)	< .001
HSCL-10, *M* (*SD*)	1.70 (0.59)	1.98 (0.67)	< .001
HSCL-5, *M* (*SD*)	1.87 (0.74)	2.23 (0.80)	< .001
Major depressive episode (MDE)	9.8%	16.9%	< .001
Generalized anxiety disorder (GAD)	8.0%	15.7%	< .001
MDE or GAD	13.1%	23.8%	< .001

For the HSCL-25, the optimal cut-off values for identifying cases of MDE or GAD were 1.96 for males and 2.20 for females. There was a good balance between sensitivity (0.92 for males and 0.89 for females) and specificity (0.83 for both males and females), and the Youden Index was acceptable for both males (0.74) and females (0.72). The PPV and NPV for males were 0.45 and 0.99, while the corresponding numbers were 0.62 and 0.96 for females (see Table 4 and Table 5).

**Table 4:** Optimal Cut-Off Values for Females for HSCL-25, HSCL-10, and HSCL-5 for Major Depressive Episode (MDE) and Generalized Anxiety Disorder (GAD) Assessed by CIDI

HSCL version and CIDI diagnosis	Cut-off value	Youden Index	Accuracy	Sensitivity	Specificity	PPV	NPV	AUC
HSCL-25								
MDE or GAD	≥ 2.20	0.72	0.85	0.89	0.83	0.62	0.96	0.94
MDE	≥ 2.28	0.74	0.84	0.92	0.82	0.51	0.98	0.94
GAD	≥ 2.08	0.64	0.76	0.91	0.73	0.38	0.98	0.90
HSCL-10								
MDE or GAD	≥ 2.30	0.70	0.83	0.88	0.81	0.60	0.96	0.93
MDE	≥ 2.44	0.73	0.86	0.88	0.85	0.55	0.97	0.93
GAD	≥ 2.30	0.63	0.77	0.88	0.75	0.39	0.97	0.89
HSCL-5								
MDE or GAD	≥ 2.75	0.68	0.86	0.81	0.87	0.67	0.94	0.91
MDE	≥ 2.75	0.69	0.83	0.86	0.83	0.50	0.97	0.91
GAD	≥ 2.80	0.64	0.82	0.83	0.81	0.45	0.96	0.89

*Note.* CIDI = Composite International Diagnostic Interview; HSCL = Hopkins Symptom Checklist; MDE = Major Depressive Episode; GAD = Generalized Anxiety Disorder; PPV = Positive Predictive Value; NPV = Negative Predictive Value; AUC = Area Under the ROC Curve.

**Table 5:** Optimal Cut-Off Values for Males for HSCL-25, HSCL-10, and HSCL-5 for Major Depressive Episode (MDE) and Generalized Anxiety Disorder (GAD) Assessed by CIDI

HSCL version and CIDI diagnosis	Cut-off value	Youden Index	Accuracy	Sensitivity	Specificity	PPV	NPV	AUC
HSCL-25								
MDE or GAD	≥ 1.96	0.74	0.84	0.92	0.83	0.45	0.99	0.94
MDE	≥ 2.00	0.76	0.84	0.94	0.82	0.37	0.99	0.95
GAD	≥ 1.96	0.69	0.80	0.91	0.79	0.27	0.99	0.92
HSCL-10								
MDE or GAD	≥ 2.10	0.72	0.82	0.91	0.81	0.42	0.98	0.94
MDE	≥ 2.30	0.74	0.87	0.87	0.87	0.41	0.98	0.95
GAD	≥ 2.30	0.68	0.85	0.83	0.85	0.32	0.98	0.91
HSCL-5								
MDE or GAD	≥ 2.25	0.68	0.83	0.85	0.82	0.42	0.97	0.92
MDE	≥ 2.25	0.68	0.81	0.88	0.80	0.32	0.98	0.92
GAD	≥ 2.20	0.64	0.74	0.92	0.72	0.22	0.99	0.91

*Note.* CIDI = Composite International Diagnostic Interview; HSCL = Hopkins Symptom Checklist; MDE = Major Depressive Episode; GAD = Generalized Anxiety Disorder; PPV = Positive Predictive Value; NPV = Negative Predictive Value; AUC = Area Under the ROC Curve.

For the HSCL-10, the best cut-off values to identify cases of MDE or GAD were 2.10 for males and 2.30 for females. The balance between sensitivity (0.91 for males and 0.88 for females) and specificity (0.81 for both sexes) was good. The Youden Index was satisfactory for both males (0.72) and females (0.69). The PPV and NPV for males registered at 0.42 and 0.98, respectively, and for females, these values were 0.58 and 0.96.

For the HSCL-5, the optimal cut-off values to identify cases of MDE or GAD were 2.25 for males and 2.75 for females, and similar to the two longer HSCL iterations, the balance between sensitivity and specificity was notably good (see Tables 4 and 5 for details). The Youden Index showed satisfactory results for both males and females at 0.68, and the PPV and NPV for males stood at 0.41 and 0.97, respectively, whereas for females, these figures were 0.65 and 0.94.

The corresponding values for only MDE and only GAD were relatively similar to those of MDE or GAD. The same optimal cut-off values for all HSCL iterations were replicated in bootstrapped analyses with 1,000 runs.

As also detailed in Table 6, when using the optimal cut-offs, all three HSCL iterations were associated with a marked overestimation of the prevalences, according to the self-administered CIDI. For example, the 30-day prevalence rates of MDE or GAD were 13.1% for males and 23.8% for females. However, using the optimal HSCL-25 cut-offs, these rates increased to 26.9% for males and 33.8% for females, respectively.

**Table 6:** Prevalence of Mental Disorder According to CIDI and the Optimal HSCL Cutoffs

HSCL version and CIDI diagnosis	Males		Females	
	CIDI	HSCL^a^	CIDI	HSCL^a^
HSCL-25				
MDE or GAD	13.1%	26.9%	23.8%	33.8%
MDE	9.8%	25.1%	16.9%	30.2%
GAD	8.0%	26.9%	15.7%	39.3%
HSCL-10				
MDE or GAD	13.1%	28.3%	23.8%	35.2%
MDE	9.8%	20.6%	16.9%	27.2%
GAD	8.0%	20.6%	15.7%	35.2%
HSCL-5				
MDE or GAD	13.1%	26.6%	23.8%	28.7%
MDE	9.8%	26.6%	16.9%	28.7%
GAD	8.0%	33.3%	15.7%	28.6%

*Note.* CIDI = Composite International Diagnostic Interview; HSCL = Hopkins Symptom Checklist; MDE = Major Depressive Episode; GAD = Generalized Anxiety Disorder.

^a^Estimated prevalences based on the optimal HSCL cut-off.

## Discussion

The present large-scale study of students in higher education employed a recently adapted self-administered psychiatric diagnostic survey (CIDI 5.0) to investigate the efficacy of three iterations of the widely used HSCL scale in detecting cases of GAD or MDE, with an emphasis on potential sex-specific cut-off values. Our results show that all three versions of the HSCL discern relatively well between students afflicted with and without generalized anxiety disorder (GAD) or major depressive episode (MDE), and that different cut-offs for males and females should be used to ensure a good balance between sensitivity, specificity and overall accuracy.

A significant finding in this study is the introduction of new, sex-specific cut-off values for all HSCL iterations. These values diverge from those in earlier validation studies, which might have lacked the statistical power to evaluate distinct cut-offs for males and females. While the conventional cut-off value of 1.75 for the HSCL-25 has been consistently used for both sexes for years, the present study indicates that adopting slightly elevated cut-offs enhances the balance between sensitivity and specificity for both males and females across all HSCL iterations. Prior research contrasting the full HSCL-25 with structured diagnostic interviews have demonstrated a sensitivity ranging from 70-88% and a specificity between 77-85% for mood disorders or depression, and 43-50% sensitivity with 83% specificity for anxiety disorders (Rodríguez-Barragán et al., 2021; Sandanger et al., 1998; Veijola et al., 2003). In the current study, both the sensitivity and specificity were generally a little higher both for MDE (sensitivity 92-94%, and specificity 82%), and GAD (sensitivity 91%, and specificity 73-79%). It is important to note that the different studies employ different anxiety and depression diagnoses.

What is noteworthy is that both the shorter HSCL-10 and HSCL-5 displayed similarly high levels of sensitivity and specificity. Although very few studies have investigated the case-detection ability of these shorter HSCL iterations, a recent study by Rodríguez-Barragán et al. found that the HSCL-5 yielded a sensitivity of 78% and a specificity of 73% for depression. Based on our dataset using the newly suggested cut-offs, we observed an even higher sensitivity and specificity for both MDE and GAD, suggesting that both the HSCL-10 the HSCL-5 may be equally good alternatives to be used in epidemiological research for the purpose of detecting probable cases of depression and anxiety. However, it should be noted that the relative differences between HSCL and CIDI prevalences were more pronounced in men. This was driven by differences in base prevalence as measured by CIDI; specifically, a relatively lower CIDI prevalence estimate is, all other things being equal (ceteris paribus), associated with a higher HSCL prevalence estimate. Another explanation for the disparities between HSCL and CIDI prevalence is that HSCL does not assess how symptoms affect daily functioning, while this is a key requirement for diagnosing a mental disorder in the CIDI instrument. This difference might partly explain why specificity was somewhat lower for GAD than for MDE. Anxiety disorders often have a more intricate relationship with functional impairment compared to depression (McKnight et al., 2016). Future studies could explore improving the HSCL by adding a measure of daily functioning. This addition could address the tool's current limitation of potentially overestimating mental health disorders due to the lack of assessment of the real-world functional impact. A revised HSCL with functional impairment questions could offer a more thorough evaluation. Alternatively, it could be used initially for screening, followed by more detailed assessments of daily functioning in those with elevated scores, ensuring a balanced approach that combines ease of use with comprehensive symptom analysis. Integrating functional impairment assessment would thus significantly enhance HSCL's diagnostic accuracy.

Surprisingly, few recent studies, both generally and specifically on university samples, have used structured diagnostic interviews, with the latest being over a decade old (Kou et al., 2012; Verger et al., 2010). The Dutch NEMESIS-3 study (ten Have et al., 2023), which monitors mental disorders in the Dutch general population, offers valuable insights. Using CIDI 3.0 (face-to-face interviews), the 12-month prevalence of any mental disorder was found to be 40% among young adults aged 18-24 years and 35% among those aged 25-35 years. Lifetime prevalence estimates were 50% and 59%, respectively. A sub-study from the 2020 HUNT study also provides relevant data (Knudsen et al., 2021). In this study, 2,154 participants from the general population were interviewed using CIDI 5.0. The 30-day prevalence of mental disorders among those aged 20-29 years was estimated to be 25.5% just before the COVID-19 pandemic. Although the prevalence estimates from NEMESIS-3 and HUNT are lower than those in our study of college and university students, they highlight the high prevalence of mental disorders among young adults.

Some methodological considerations warrant attention. This study's reliance on the standardized and validated CIDI psychiatric survey represents a significant strength. However, the shift from traditional face-to-face interviews to a self-administered electronic format in CIDI 5.0 introduces challenges, such as the need for further validation against conventional methods. Past research (Knudsen et al., 2021) indicates no significant prevalence differences between face-to-face and telephone interviews, although recent comparisons between face-to-face and web-based self-reporting of psychological functioning revealed that respondents in face-to-face settings reported slightly fewer symptoms of depression (Cohen’s *d* = 0.25) (Kocjan et al., 2023). This raises questions about the accuracy of different administration modes, particularly as previous findings suggest that young, well-educated respondents might underreport mental health issues. Assessing the reliability of the self-administered CIDI, especially in comparison to face-to-face interviews, is crucial not only for validation but also for understanding how different modes might impact mental health assessments. However, discrepancies between HSCL and CIDI prevalence estimates, particularly among male respondents, require careful interpretation. These variations underscore the need for contextual adjustments and a deeper understanding of how assessment tools could produce divergent outcomes in mental health research. The proposed cut-offs, tailored for epidemiological research in a student population with unique demographic traits, might not generalize well to other groups due to varying baseline prevalences and cultural perceptions of mental health. Therefore, while HSCL shows promise as a screening tool in a student population, its broader applicability needs further validation. Moreover, the limited differences observed in mental health problems between CIDI respondents and non-respondents, along with consistent response rates across Norwegian regions, suggest a reasonable level geographic representativeness. However, our understanding of non-responders, limited to basic demographic details, restricts our ability to fully assess representativeness against the broader Norwegian student population. Recent findings from Denmark (Lyngsøe et al., 2023), showing minor participation variations across sociodemographic groups provide some reassurance regarding the generalizability of our results.

The best approaches to determining cut-off values based on scales, like the various HSCL variants, depend on the intended purpose. If the goal is to identify individuals potentially in need of psychiatric treatment, prioritizing high sensitivity is crucial. However, when estimating the prevalence of depression or anxiety disorders within a population, striking a balance between specificity and sensitivity relative to the true prevalence of the disorder might yield more accurate results. Recommending specific cut-off values for future studies lies beyond the scope of this study. Such norms can be effectively determined only after conducting several similar studies, ideally across diverse cultures, countries, and population segments. The benefits of using different cut-off points for men and women also warrant more comprehensive evaluation, compared against the challenges posed by adding such complexity.

## Data Availability

Data are available upon reasonable request. All SHoT data set is administrated by the NIPH. Approval from a Norwegian regional committee for medical and health research ethics (https://helseforskning.etikkom.no) is a prerequisite.

## Preregistration

The study was not preregistered. The CIDI follow-up survey is registered at ClinicalTrials.gov (identifier: NCT05731102).

## Reporting Guidelines

We adhered to the STROBE (Strengthening the Reporting of Observational Studies in Epidemiology) guidelines in the design, conduct, and reporting of this study.

## Statement of the Conclusions

The study revealed high criterion validity for all HSCL versions in detecting major depressive episode and generalized anxiety disorder, suggesting their efficacy as screening tools rather than for precise prevalence estimation.
